# VirtLAB: A Low-Cost Platform for Electronics Lab Experiments †

**DOI:** 10.3390/s22134840

**Published:** 2022-06-27

**Authors:** Massimo Ruo Roch, Maurizio Martina

**Affiliations:** Department of Electronics and Telecommunication, Politecnico di Torino, I-10129 Torino, Italy; maurizio.martina@polito.it

**Keywords:** FPGA, MCU, electronics lab, virtual lab, electronics teaching

## Abstract

The recent SARS-CoV2 pandemic has put a great challenge on university courses. Electronics teaching requires real laboratory experiences for students, which cannot be realized if access to physical infrastructures is prohibited. A possible solution would be to distribute to students, at home, electronics equipment suitable for laboratory experiments, but no reasonable product is currently available off-the-shelf. In this paper, the design and development of a very-low-cost experimental board tailored to these needs is presented. It contains both programmable prototyping circuitry based on a microcontroller and an FPGA and a set of measurement instruments, similar to the ones found on a typical lab desk, such as a digital storage oscilloscope, multimeter, analog signal generator, logic state analyzer and digital pattern generator. A first board, suitable for analog and digital electronics experiments, has been designed and manufactured, and is described in this paper. The board has been successfully used in master’s degrees and PhD courses.

## 1. Introduction

Starting from the spring of 2020, the SARS-CoV2 pandemic hit China, Italy, and the rest of the world. Lockdown countermeasures were mandatory in order to mitigate virus spread among the population. This means that teaching ’in presence’ was stopped, too, in schools of every rank, from primary up to universities [1].

Current Internet capabilities allow us to overcome difficulties related to lectures, as videoconferencing is a well-established technique [2], even if there are some challenges that arise when the number of attendants goes beyond some hundreds if direct interactivity is desired (this concerns real-time lessons, and not just playing recorded videos) [3]. Nowadays, host virtualization and private or public clouds are already used inside universities, allowing us to deploy into the campus remote access to computing platforms [4], even at a supranational level [5].

Moreover, web applications allowing us to exploit interactivity in simulated environments, such as Moodle [6], are of widespread usage, and can be used to introduce exercises and simulated laboratory experiments, in which, students can interact with virtual objects, changing their parameters and simulating the behaviour of the so-modified experiments.

Electronics engineering courses are natural candidates for these kinds of web-based tools. As an example, in analog and digital electronics courses, students can design circuits, either via schematic capture or Hardware Description Languages (VHDL, Verilog, SystemC). Later, they can perform analog, digital, or mixed-signal simulations, utilizing web interfaces toward standard simulation tools (Spice, Modelsim) [7,8].

What is missing in this approach is the contact with real-world objects, which, according to us, is fundamental for the acquirement of specific engineering skills. In fact, developing design capabilities requires acquiring the following abilities:

(a) Design space exploration;
(b) Simulation of the designed system;
(c) Verification of compliance to a previously defined high-level reference model through every refinement step;
(d) Hardware verification, with limited debugging capabilities;
(e) Characterization of the hardware system.

Points (a) through (c) can be easily accomplished with the web-based methodologies described above. However, the last two items are, nowadays, not affordable at all.

First of all, it must be emphasized that simulation cannot fully substitute measurements performed on the real circuit. In fact, the major drawback of simulation performed inside courses is the lack of coverage of the tests, mainly due to student inexperience and time shortages. Therefore, hardware verification is necessary.

Moreover, the ability to diagnose real circuit faults is a typical high-value engineering skill that must be pursued. In addition, it requires skills in both real measurement instrument usage and the personal development of fault search methodologies specifically targeted toward digital or analog circuits.

Lastly, real signals are usually very different from simulated ones, sometimes in surprising ways for a student, and it is important to be able to visualize them in a realistic manner, as sampled by a real measurement instrument, including noise and other artifacts.

A possible solution to these requirements would be to give to students access to physical devices on which to perform lab work, but, at the same time, to learn measurement instrument usage, too.

In this context, there are three different possibilities:

(a) Universities buy and send to students a ‘lab kit’ built up by a set of boards suitable for lab needs. As an example, one MCU board, one FPGA board, a digital oscilloscope, a power supply, etc. This approach has a problem from the point of view of the cost, as the number of kits must be greater than the number of students that are following courses using the kits itself. In fact, in contrast to what happened with ’in presence’ labs, no sharing is possible for lab appliances, as they are physically at the home of the student. Moreover, there are logistics problems too due to the complexity involved in the delivery of kits to students before the course starts and the collection of them after the course ends. Furthermore, this problem worsens if students are located in different cities or countries (at Politecnico di Torino, one half of the students come from different regions, and one tenth from different countries).
(b) Universities suggest the above kit to students (like what happens for a course text book). Of course, this solution is affordable only if the total kit is low and acceptable for the student’s balance. This is the problem. In fact, it is quite easy to find low-cost experimental boards that can be used to implement the experiment: typically, they are MCU-based (Arduino, Nucleo), with prices in the range of USD 10–20. However, there are very few low-cost boards suitable for programmable logic development (at around USD 100 each), and, worst of all, it is nearly impossible to obtain a set of low-cost measurement instruments that are suitable for reasonably sophisticated experiments (the minimum is around USD 200).
(c) Universities install on-premise measurement instruments, and boards host experiments, giving access to them via the Internet. The cost is similar to approach (a), but the drawback is that students do not have physical access to the components.

Some examples of the just-described approaches are:

- EPFL-choice b-Embedded systems master course-MSP432P401R-LaunchPad, DE0-nano, DE0-nano-SOC, DE1-SOC with small piggybacks [9,10,11];
- Politecnico di Milano-choice b-STM32 Nucleo with custom developed I/O piggyback [12];
- Politecnico di Torino-choice a-Electronics Engineering master course-Breadboard with off-the-shelf passive and active components, and Analog Discovery acquisition system [13];
- Mobile Studio-choice b-Custom board with emulated instruments to be integrated with other boards to host experiments [14];
- Universite de Pau et des Pays de l’Adour-choice c-High cost per seat [15];
- Universitat Oberta de Catalunya-chioice b-No user circuits, only simple prototype board [16].

The main reason for why no suitable solution has been found on the market is mainly related to the fact that systems building a kit are not developed with teaching in mind. They are general purpose boards designed for small-scale prototyping or for technology evaluation. As such, they have much more hardware than needed, i.e., their cost is not at a minimum. Equally, measurement instruments are true, complete, and sophisticated devices with overabundant features, i.e., again, they are too expensive.

The basic idea to fill the above gap is the development of a technology—a platform, specifically targeted toward teaching labs, aiming to minimize the unit cost but fulfilling electronic lab requirements. It will be realized as a mix of hardware/software components and could allow for three different usage models:

- The students use it at home. No additional hardware must be required, except for the experiment platform itself and a personal computer (laptop or desktop).
- The students use it in the campus labs. This can be a duplication of existing lab equipment, but this choice allows for the use of the same course material in different situations, i.e., remote labs or ’in presence’ ones, and to avoid biases between on-site and off-site students.
- The student is at home, but can have Internet access to the experiment deployed inside university labs. A critical point is to mimic, as far as possible, the same user interface as in preceding use case in order to maintain a uniform usage experience.

Three fundamental differences must be emphasized when comparing the proposed solution to existing ones:

- Co-existence inside the same board of both measurement instruments (oscilloscopes, waveform generators, logic state analyzers, etc.) and experimental circuits (microcontrollers, programmable logic, analog and digital I/O).
- Cost minimization through strict performance tailoring to teaching activities.
- Size and robustness optimization, as needed for a teaching platform used by inexperienced users too.

Moreover, as the developed system will be dedicated to teaching, the design methodology requires starting from the analysis of lab experiences common in the target courses of the experimentation.

In the following sections, an expanded version of [17], a possible solution is proposed. First of all, a generic architecture is described, and then a first case study implementation is presented.

## 2. System Architecture

The basic idea is to develop a hardware device with two sections. The first one, called the "master area", is designed to resemble a typical lab desk, integrating the functionalities of a digital storage oscilloscope (DSO), a standard multimeter, and a programmable analog signal generator. Moreover, in this section, the teacher can load special purpose devices, e.g., test beds for the lab experience carried out by students. The second section ("user area") must instead be the equivalent of a prototype board, on which, students can carry out the experiment itself.

The board must be easily connected to a computing device (PC, Raspberry, etc.), where high-cost computational tasks can be performed. These tasks are, as an example, signal analysis algorithms (FFT, noise filtering, digital protocol analysis), data presentation to the user, and virtual instrument controls.

The integration of custom hardware and software running on the computing device is the key to minimizing the cost of the overall system. In fact, repetitive costs (hardware) are minimized at the expense of designing PC software, but the latter can be developed through an Open Source model. This choice introduces a further possibility, i.e., to involve computer science students in the development of this software, too. This fulfills another teaching activity, well-integrated inside an IT degree, even if not strictly required by initial specifications.

In this architecture, students can access the experimental board either directly or through the Internet, where a Raspberry PI or a laboratory server exposes the user interface.

**Figure 1:**
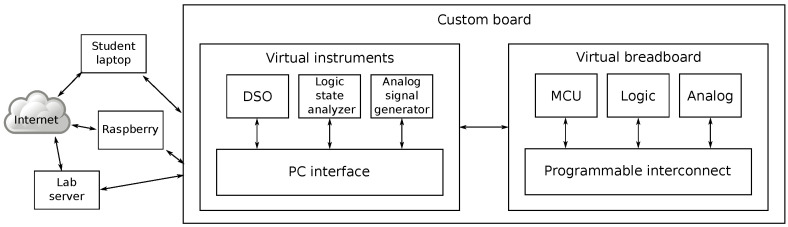
Basic architecture of the system.

The overall architecture is depicted in Figure 1.

## 3. Design of a First System

To assess the feasibility of the proposed approach, a first system was designed, specifically aimed toward electronics engineering courses in a master’s degree. Target courses are related to embedded systems design, low-power digital electronics, programmable logic, and bio-medical electronics systems. Skills acquired by students are mainly in the field of HDL design methodology, MCU hardware/software integration, embedded systems firmware development, analog and digital data acquisition and processing techniques, and hardware/software low power design.

The design started collecting a set of all of the lab experiences currently carried out in the above courses using off-the-shelf instruments and platforms. From these, a common base of required hardware facilities was extracted, resulting in an inventory of mandatory features. This way, the board guarantees that it will be capable of implementing all of the required lab experiments.

An extract of the main experiments is as follows:

- Generation of digital waveforms with I/O ports of a microcontroller. Timing comparisons for polling, interruption, and hardware timer-assisted generation.
- Audio signal acquisition from digital microphones. Signal reconstruction with MCU and FPGA. Sound event detection. Sound source direction detection through stereo microphones.
- Acquisition of real time data from three-axis accelerometers. Position calculation via MCU and FPGA. Graphics representation in an FPGA-based frame buffer.
- Implementation of serial communication peripherals IP and testing on FPGA (I2C, RS232).
- Implementation of parallel bus handshake protocols master and slave (synchronous, semi-synchronous, asynchronous).
- Implementation of hardware computing accelerators (FIR, IIR, FFT, AI). Power consumption optimization techniques.

Thereafter, features not substantially increasing the total cost of the board are introduced in order to maximize the flexibility to implement new experiences. As an example, if the maximum number of digital I/O pins needed to implement existing labs was 13, and supposing that I/O ports are commonly available with 8 bit parallelism, it will be straightforward to have 16 I/O pins, with a negligible cost increment.

**Figure 2:**
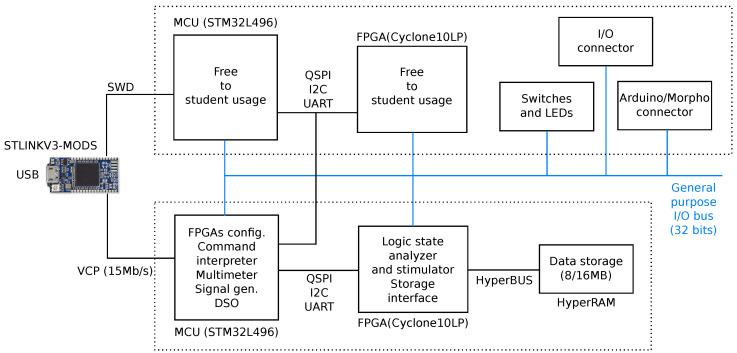
Block diagram of the designed board.

A high-level block diagram of the board is visible in Figure 2.

Three main blocks are visible: the user area (upper half), the master area (lower half), and the USB interface (STLinkV3MODS, at extreme left). The master and user sections are linked by a 32 bit general purpose bus, which is freely usable by experiments. The same bus is exposed through I/O pins, too.

Not visible in the diagram is the power supply section, which is needed to feed the right voltages and currents to on-board devices.

In the following subsections, a detailed description of each block is reported.

### 3.1. Master Section Specifications

The design of this area must be carried out by exploiting a trade-off between costs and performances. The first step is to then define virtual instrument specifications, both in terms of the type and the performance.

It is clear that, given a target board cost of less than USD 100, it is impossible to use sophisticated analog circuits, and mixed-signal MCU capabilities must be exploited as much as possible. As a consequence, specifications must be compatible with standard low-cost commercial hardware availability. The design process will then use the identified requirements as hints, and not as mandatory specifications. Several possible virtual instruments were identified, and tentative specification was derived, as described in the following Section 3.1.1, Section 3.1.2, Section 3.1.3, Section 3.1.4 and Section 3.1.5.

#### 3.1.1. Digital Storage Oscilloscope

The DSO implemented will have limitations due to low-cost analog front-end performances (low precision, low sampling frequencies, etc.). However, this limitation can be converted to an advantage. In fact, having limited capabilities can allow the student to easily experience side-effects derived from system limitations. As an example, the injected switching power supply noise on DSO analog input channels can demonstrate the importance of CMRR at high frequencies, or the limited sampling rate can be used to directly show aliasing effects when the Nyquist criteria are violated.

In addition, a reduced instrument bandwidth is not a real problem for university courses. Typical laboratory experiences with analog signals are performed with frequencies from DC of up to 100 kHz. In addition, digital signals commonly used on prototype boards, too, have a bandwidth in the order of 100 kHz. The I2C standard, RS232, and SPI are typically used in teaching labs at those frequencies.

Then, it was considered reasonable to have the analog section have a bandwidth of less than 1 MHz.

Regarding input dynamics, as the input signals are mainly derived from the board itself, having a very wide range of accepted input voltages was not needed, limiting them to the power supply range of the system itself.

To simulate the behavior of a real oscilloscope in an effective way, including probes, a compensated divider with an attenuation of 10 is needed, followed by a rail-to-rail I/O operational amplifier with a gain of 10. The degree of compensation must vary by the user in order to be able to show under- and over-compensation effects on input signal visualization.

Two analog voltage input channels are the acceptable minimum to at least be able to compute transfer functions and component trans-characteristics, and to perform similar experiments.

The developed system can be used in courses that talk about low-power firmware and hardware design, too. Then, it could be interesting to have the capability on-board to perform supply current measurements on user section devices. This is a feature not often found in commercial instruments due to the need to break power supply lines feeding the device that is being tested. However, as user devices and virtual instruments are hosted by the same board, it is feasible to put some current sensors on supply rails. Ideally, every supply rail feeding devices in the user section must be instrumented, allowing for the separation of the integrated circuits core supply current from the I/O one. Acquired currents must be converted to voltages and sent to auxiliary channels of the DSO. To be able to correlate voltage input channels to current ones, a common trigger mechanism and common timescales must be provided.

Standard DSO triggering schemes must be provided (Auto, Normal, Single, Stop) with freely configurable edge polarity, too. Any input channel can be selected as a trigger event source, and an external digital trigger input source must be available, too, to synchronize measurements to digital hardware or software events.

No visualization interface is needed, and just raw data must be stored. Then, they can be sent to a host PC for data processing and viewing.

#### 3.1.2. Multimeter

A digital multimeter is a useful tool to perform accurate analog measurements on a prototype board. However, as a two-channel acquisition system is already present to implement the digital storage oscilloscope, it seems reasonable to use it directly as a multimeter. In fact, the higher precision required can easily be exchanged with the reduced bandwidth. In other words, a low resolution, moderate bandwidth, and noisy signals acquired by the DSO can be numerically processed through digital filters to improve the signal-to-noise ratio at the price of a low-pass filter. There is no need to perform this kind of processing task on-board, as it can be easily accomplished by the host interface. Moreover, the fact that there are two DSO voltage input signals means that differential measurements can be easily performed by just subtracting, sample-by-sample, the two input data streams. As a consequence, no further design was needed, despite the introduction of a virtual multimeter in the specifications.

#### 3.1.3. Analog Signal Generator

A second mandatory virtual instrument is an analog signal generator. Typical waveforms available in commercial instruments include sine, triangular, and rectangular ones. To maximise flexibility and performances, an arbitrary waveform generator, generating outputs from stored digital samples, is needed. The samples can either be generated locally or transferred from the external controlling host.

One channel is mandatory, but two output channels are desirable, if possible, to allow for the generation of different waveforms injected in the same circuit (e.g., a high-frequency carrier, and a low-frequency modulation signal sent to a processing block, or quadrature sine waves used for QAM, PSK, etc.). If a second output channel is introduced, it must be able to be freely running, or synchronized to the first one, in order to maintain precise phase relationships.

From the point of view of generated frequencies, it seems reasonable to have bandwidths similar to DSO analog input channel ones, i.e., approximately 1 MHz.

#### 3.1.4. Logic State Analyzer

A logic state analyzer is a commonly used analysis and debug tool used on digital circuits. Its applications range from the visualization of digital communication serial or parallel protocols to the study of the evolution of a finite state machine.

In a digital design course, this tool can simplify the detection of design flaws, such as the misbehaviour of control units or deviation from standards for communication protocols. Moreover, a decoding visualization interface can make it easier for the interpretation of a complex serial bus transaction, such as for I2C or SPI and their derivatives.

Nevertheless, it is not common to have logic state analyzers on laboratory benches due to the high cost of commercially available ones. This cost is mainly related to the need to have interfaces for different voltages, which vary according to the logic standard considered.

However, in this case, as the signal entering the instruments is typically generated by the board itself, there is no need to introduce expensive multichannel level translators, thus greatly simplifying the circuitry needed to implement a logic state analyzer.

From the point of view of required performances, it is reasonable to require sampling rates far higher than DSO ones in order to be able to use the logic state analyzer profitably in mixed-signal designs, where the digital data flow runs at word rates higher than analog sampling ones. Glitch detection must be implemented on each channel, too, which is a useful feature in static and dynamic hazards laboratory experiments.

A flexible triggering schema must be implemented in order to be able to check several inputs at once and to evaluate them against a digital pattern. If feasible, a triggering schema based on the detection of a specific sequence of digital patterns must be introduced too.

Raw acquired data must be stored locally, and later sent to the host PC for analysis and visualization.

Lastly, an eventual protocol-decoding capability, desirable from the point of view of data visualization, was delegated to the host controlling the board.

#### 3.1.5. Digital Pattern Generator

This virtual instrument has a twofold application in laboratory experiments carried out on the designed board. First, it can be used by the teacher to generate test patterns, sent to the circuits designed by students, and implemented in the user section. As a second usage, it can be used by students to learn and check their ability to write adequate test patterns to verify a defined hardware behaviour, eventually computing the test coverage and reliability, too.

Given the unpredictability of use cases, it is preferable to have a simple table-driven pattern generator. In the case of complex protocol generation, the task of raw pattern generation is left to the host PC, which will send tabular data to the board.

The pattern generation speed must be comparable with the bandwidth of the logic state analyzer. In fact, it is not really useful to generate signals that cannot be acquired for the logic state analyzer, as, in this case, even the logic implemented in the user section to treat these signals would not be easily verifiable.

### 3.2. Master Section Implementation

Given the above specifications, the decision to design a four-chip solution was made, allocating virtual instruments according to hardware capabilities. In more detail, this area contained four main devices:

- Master MCU.
- Master FPGA.
- Master QSPI flash.
- Master HyperBUS RAM.

#### 3.2.1. Master MCU

The master MCU is a mixed-signal microcontroller based on a 32 bit ARM Cortex-M4 microprocessor. The chosen device was a low-power MCU from STM (STM32L496VET). Given the high count of interconnections required, but also the need to be able to eventually repair the board in-house, a 100-pin quad flat pack (QFP) derivative was chosen. In fact, the usage of ball grid array (BGA) devices would have required complex and costly equipment for an eventual replacement. This is a possible, even if not probable, event, as some pins of the MCU, as described later (Section 3.5), are accessible through external I/O connectors. The main data of the selected MCU were:

- Core clock frequency: 80 MHz;
- Flash memory: 512 kBytes (two equally sized independent banks);
- RAM memory: 320 kBytes;
- QUADSPI high-speed interface;
- Multiple communication interfaces (SPI/UART/I2C/CAN);
- One USB-OTG interface;
- Five Msps, 12 bit, triple ADCs, with hardware oversampling;
- Two channels, 12 bit DAC;
- Two operational amplifiers;
- Two comparators;
- Fourteen-channel DMA controller;
- A 1.7 V to 3.6 V power supply.

The first task of the master microcontroller is the implementation of the low-speed portion of virtual instruments (waveform generator and digital storage oscilloscope).

The DSO was implemented through two internal ADCs of the MCU. They continuously sample analog data on the input channels and store, via DMA, the results in a ring buffer. Whenever the selected trigger condition is met, the current position of the write pointer in the buffer is saved, and data sampling continues to read a further half of the buffer size. With this technique, the circular buffer will contain exactly half of the samples in the past, and half in the future, around the trigger sample. The input channels of the DSO were as follows:

- Voltage input channel 1 (compensated probe);
- Voltage input channel 2 (compensated probe);
- Supply current of user FPGA I/O (3.3 V);
- Supply current of user FPGA PLL (2.5 V);
- Supply current of user FPGA core (1.2 V);
- Supply current of user MCU (3.3 V).

**Figure 3:**
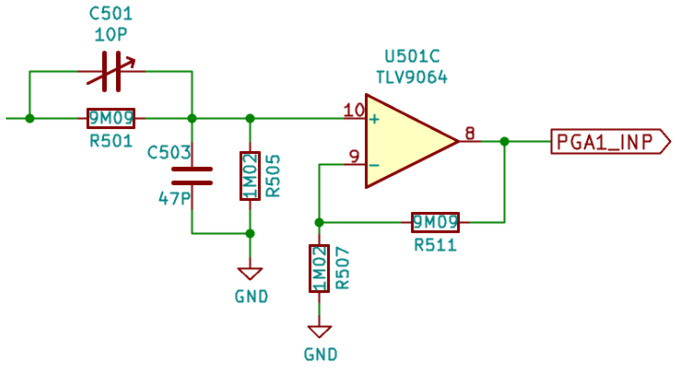
Schematic of a DSO voltage input channel.

In Figure 3, the schematic of a voltage input channel is depicted (two identical instances are present on the board). The operational amplifier used was a low-cost rail-to-rail I/O CMOS type, with no phase reversal and a fast recovery on overload. Due to its 10 MHz unity gain bandwidth, a −3 dB bandwidth of approximately 1 MHz was obtained for the front-end. The capacitor C501 on the input can be trimmed by the student to compensate for the voltage divider, and to obtain a flat response in the pass-band, independent of the source impedance. The output of the amplifier fed a programmable gain amplifier inside the microcontroller, set to unity gain. If needed, it can be configured for higher gains, up to 16, but at the cost of reducing the available bandwidth, as the internal operational amplifier has a unity-gain frequency of 1.6 MHz.

**Figure 4:**
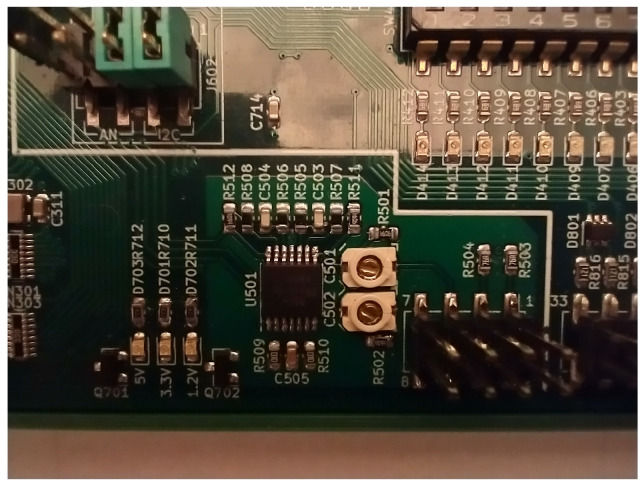
Layout of the DSO voltage input channel. The two white rectangular components in the center are the variable capacitors used to compensate for the voltage divider.

In Figure 4, the physical implementation of the two input channels is visible. The black IC in the middle of the photo is a TLV9064 quadruple operational amplifier used as the analog front-end. Immediately on the right, the two rectangular white devices are variable capacitors, trimmable with a screwdriver. Input signals to the probes are connected to the nearby gold plated header. Very short distances are mandatory, so as to reduce costs; there is no electric shield to reduce noise coupling.

**Figure 5:**
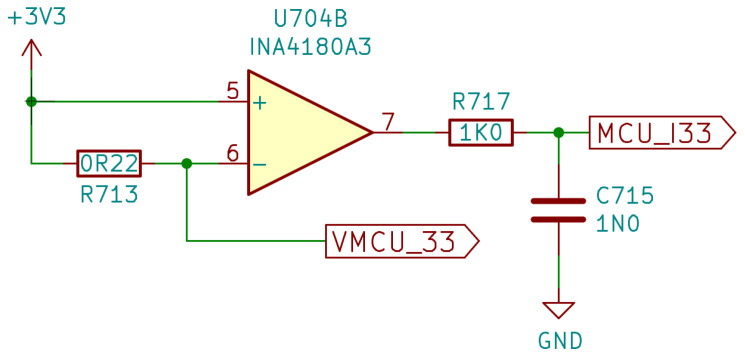
Schematic of a DSO supply current input channel.

In Figure 5, the schematic of a supply current sensor circuit is drawn (four instances are present on the board). It is built around a current sense amplifier, optimized to sense high-side differential voltages. The resistor R713 is the shunt element, inserted between the 3.3 V power supply, and the supply rail of the user MCU (VMCU_33). The total trans-resistance gain is given by the value of the shunt resistor multiplied by the voltage gain of the INA4180A3 (100 V/V) and must be adapted according to the maximum current that must be measured, and the maximum voltage drop acceptable by the device fed by this circuit. In fact, whereas MCUs can accept wide variations in the power supply voltage (of the order of 1 V), FPGA core power requirements are far more stringent (50 mV, including power supply tolerances). Table 1 summarizes the design characteristics of the DSO analog front-end.

The six input channels were converted using two different ADCs, connected in a synchronized master/slave configuration. At the selected sampling rate, two conversions of paired channels were initiated at the same time. Each channel was converted eight times, and two average values were computed in the hardware. When the input pair conversion was terminated, a DMA access moved the two 12 bit values to the circular buffer with an atomic operation. This technique guarantees minimum time lag between paired channels, and it is therefore ideal to use channel 1 and channel 2 as a single differential channel, rejecting common mode environmental noise, too. The sampling time was decided by a hardware timer, that, when a timeout elapses, triggers the conversion of three sample pairs. The firmware must only check data values, via an interrupt service routine (ISR), for input trigger detection at the end of the conversion batch.

It must be noted that the oversampling factor was not fixed, and coud be changed according to specific lab experiences. As an example, the default 8× oversampling was not suitable for completely eliminating aliasing effects, as the input amplifier had a bandwidth of 1MHz. However, this is a desired feature, as it is able to show to students the effects of Nyquist criteria violation on a sampled signal.

The waveform generator was implemented in similar way, through a circular buffer inside the microcontroller RAM, which directly fed the two channels of the internally available DAC. Data movement from the buffer to DAC registers was performed by a DMA access, triggered by a hardware timer. No microprocessor software instruction must be executed to generate the output signal after hardware initialization.

A second task of the master microcontroller is to take care of host-to-board communication, too. To maximize data transfer speeds, the USB interface was selected. The MCU has integrated hardware support for the USB 2.0 Full Speed protocol. To simplify the firmware development on the board side, and to avoid device driver complexity on the host side, the decision to declare the board as a communication device was made, compliant with the CDC-ACM class standard. The bandwidth available on the communication channel was approximately 10 Mb/s. In other words, when attached to a host, the board appeared as a simple serial port, but with a baud rate far higher than the speed available in physical serial devices. Moreover, the CDC-ACM class is supported in every operating system currently available, without additional software installations, simplifying client software development and deployment. The USB connection was physically implemented with a USB-C receptacle, chosen for the superior mechanical and electrical characteristics, with respect to mini- or micro-USB connectors.

A third task must be accomplished by the master microcontroller. In fact, as discussed later, high-speed digital virtual instruments (logic state analyzer and digital pattern generator) were implemented on the master FPGA. The MCU must then manage host commands directed to this device and configure FPGA-hosted logic. Moreover, it must be possible to transfer large amounts of data to and from these virtual instruments. To accomplish this task, a high-speed hardware QUADSPI channel was used, allowing us to move data at rates of up to 40 MBytes per second. Lightweight accesses can be easily obtained, as the selected microcontroller can use a dedicated DMA channel to access QUADSPI external peripherals if they are mimicking standard memory behaviour.

The presence of master and user FPGAs implies the need to configure them after board power-on, as RAM-based FPGAs are inherently volatile, losing their internal state if supply voltage is removed. Flash-based FPGAs are not suitable in this design due to their higher unit cost for the same logic complexity. Configuration data were generated by a CAD tool used to synthesize logic derived from a hardware description language (HDL) or schematic-based design. Usually, the CAD tool runs on the host device, and the configuration data are contained in a file. The data size was approximately 6 Mbit. This file must first be transferred on-board to a temporary storage through the USB-based command interface described above, and then, after mandatory sanity checking, it must be sent to the target FPGA. This approach is not optimal, because the configuration of the master FPGA is not frequently changed, as it just contains a virtual instruments description, and it is a waste of time to transfer it every time from the host to the board. Moreover, the command channel has limited speed, and the transfer of FPGA configuration data can require several seconds. To overcome this problem, an QSPI flash was added to the design, able to store up to eight different FPGA configuration data files. At power-up, the master MCU checks the content of the QSPI flash at default positions, i.e., index 0 for the master FPGA and 1 for the user one. If a valid configuration data file copy is found inside the QSPI flash, it is directly sent to the target FPGA, configuring it in less than 100 ms. This will be the preferred configuration procedure for the master FPGA. On the other hand, the user FPGA configuration is frequently changed, and so the slower method using the command interface will be used.

Even if not specifically required by the specifications, it was decided to directly attach 10 digital I/O pins of the MCU to the less significant bits of the general purpose digital I/O bus shared among the user and master section. In more detail, PORT E of the master microcontroller, bits 9 to 0, were used. They can be freely configured as digital inputs or outputs. Alternatively, they can be used as hardware timer inputs or outputs.

Lastly, two dedicated communication channels connect the master microcontroller to the user section. They are internally linked to the I2C, UART, and CAN master microcontroller peripherals. Four low power LED’s are connected to four output pins of the master MCU to simplify firmware debugging and monitoring.

#### 3.2.2. Master FPGA

An Intel Cyclone 10 LP low-power FPGA with up to 25 k LEs was implemented in a hardware high-speed portion of the virtual instruments. As an example, it samples digital input channels of the logic state analyzer, generates digital sequences for the digital pattern generator, and stores them into the high speed RAM storage.

To maximize usage flexibility, all of the 32 bits of the general purpose I/O bus are connected to the master FPGA. They can be freely shared between the logic state analyzer and the digital pattern generator.

It works as a bridge, too, between the MCU and the RAM storage. This way, the MCU can overcome limited internal memory availability if additional storage is required for specific virtual instruments or complex data processing tasks. The FPGA can host teacher-provided custom test benches, too.

Four low-power LED’s are connected to dedicated FPGA pins. They are useful for visually checking the status of the master FPGA.

#### 3.2.3. External Storage

Even if both the master microcontroller and the master FPGA contain memories, it was decided to add dedicated external storage to increase the buffer depth of virtual instruments and to provide additional support for specific functions.

An 8 or 16 MBytes RAM was provided as a generic data store. Due to limitations on costs and package pins, a HyperBus device was chosen to be attached to the master FPGA. A sustainable bandwidth of more than 100 MBytes per second is affordable due to a DDR 8 bit data bus. The main usage of this device is for the storage of digital samples acquired from the logic state analyzer, or for patterns to be sent to the digital pattern generator.

A second storage device is represented by an 8 MBytes QSPI flash memory. It is directly attached to the master microcontroller QUADSPI interface, and its main usage, as described in Section 3.2.1, is to save frequently used FPGA configurations. Regardless, there is no drawback to allocating different contents on it in future releases of the master firmware.

### 3.3. User Section Specifications

The user section must be designed in such a way as to allow students to carry out laboratory experiences related to the arguments treated in the courses selected at the beginning of Section 3.

An FPGA is then mandatory in order to be able to implement and test HDL-specified digital designs.

A microcontroller is mandatory, too, as embedded system courses need it to experiment with user firmware development.

Hardware/software integration can be exploited using the microcontroller connected to digital logic inside the FPGA. This logic can be developed by students, too, or simply designed by teachers if HDL design knowledge is not relevant for the course.

To increase flexibility, there must be provisions for interfacing to external off-the-shelf commonly used blocks, e.g., RF transceivers, I/O expanders, digital or analog audio circuits, microphones, temperature and humidity sensors, accelerometers, etc.).

Mixed signal hardware development and related firmware programming can be exploited if the microcontroller used in the user section is a mixed-signal one, and if analog signals are available on external connectors.

As the developed board must be able to work as a standalone system, some simple user interface devices (buttons, LED’s) must be included to allow for students to interact with the firmware running on the user microcontroller and/or the logic configured inside the FPGA.

### 3.4. User Section Implementation

The user area is implemented with a microcontroller and one FPGA, identical to the ones used in the master section. The fact that they use perfectly identical components gives significant advantages in term of cost reduction due to the minimization of different part numbers on the board. Moreover, the knowledge acquired for the master section design can be applied immediately to the user section design, reducing the development time.

To obtain maximum flexibility in connecting devices, an Arduino UNO R3 compatible connector was introduced. This choice allows us to seamlessly connect any existing shield from the Arduino world to the VirtLAB board. Some jumpers allow us to connect Arduino signals to different user MCU pins in order to maximize an efficient usage of the hardware peripherals of the microcontroller.

Analog blocks present inside the user microcontroller are connected to the external world. Two operational amplifiers and one comparator are fully connected to external signals. As both inputs and the output of the components are connected, an external feedback network can be realized in order to implement simple amplifier or filter stages. Two DAC output channels are available, too, buffered through two voltage followers to minimize load effects.

The user interface required by specifications was implemented with the following devices:

- Four low-power green LEDs attached to dedicated pins of the user FPGA.
- Four low-power green LEDs attached to dedicated pins of the user microcontroller.
- One four-digit, seven-segment LCD display. It is directly attached to the user MCU, as this one contains an LCD driver, easily managed through register mapped bits (one bit per segment).
- Eight small switches, attached both to the MCU and the FPGA. To have immediate feedback on the effective switch position, every switch is connected to a yellow LED, which is turned on if the switch is in the “on” position, corresponding to a logic ‘1’ on the corresponding input.

Additional connectivity is available through a USB-C receptacle, connected to the user microcontroller, and freely available to students.

### 3.5. Connectivity

In this section, board connectivity is summarized, with reference to both internal and external connections.

#### 3.5.1. External Connections

**Figure 6:**
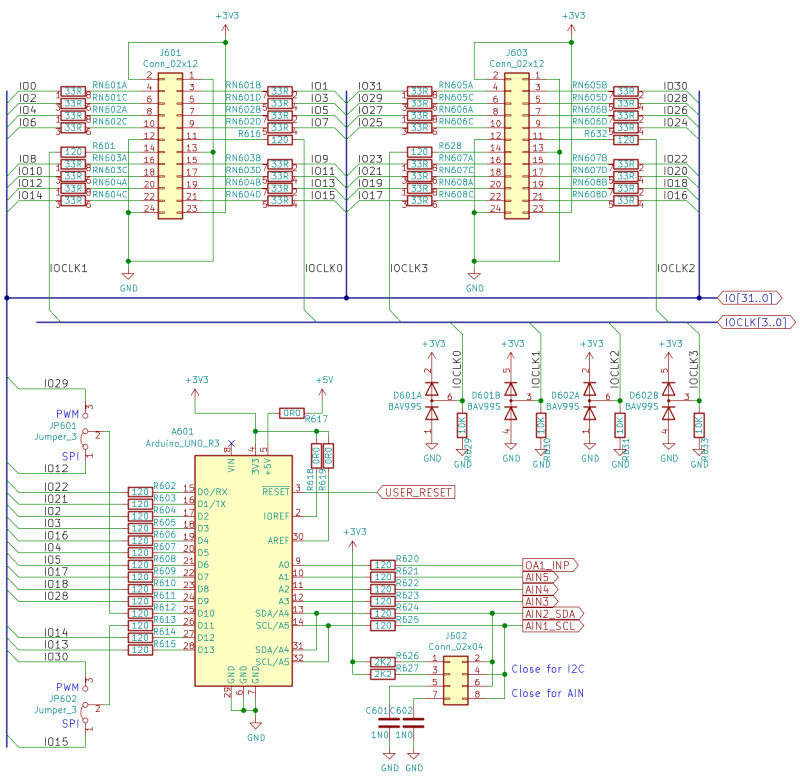
Digital I/O connectors.

There are mainly two different types of external connections: digital and analog ones. Digital connections are mainly represented by the 32 bit digital I/O bus, common also to both user and master sections. Added to these, there are four input clocks to drive the internal PLL of the user FPGA and the digital signals of the Arduino connector. Figure 6 shows the available signals.

The two top connectors (J601 and J603) are standard 2.54 mm double row headers. The left bottom connector (A601) is the Arduino one. To reduce the possibility of failures due to I/O signals driving mistakes exceeding specifications, two levels of protection are introduced: first, a resistor is connected in series to each I/O pin, and, second, two protection diodes are connected to limit voltage excursions on internal board signals. This protection is not able to sustain a permanent short-circuit to a low-impedance high-voltage source, but it gives a reasonable safety against common user mistakes.

**Figure 7:**
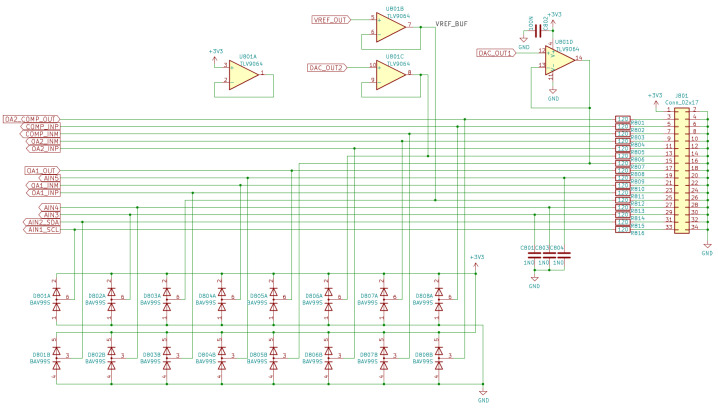
Analog I/O connectors.

Analog external connections, visible in Figure 7, are only limited to the analog blocks contained in the user microcontroller. They are the two DAC outputs, buffered by two unity-gain voltage followers, two operational amplifiers, and one comparator. Due to restrictions on the number of available pins, the comparator and the second operational amplifier outputs share the same external connections. There are also five unbuffered analog inputs, directly connected to the user microcontroller internal ADCs. Analog inputs are protected against connection mistakes with the same safety circuit used for digital I/O channels. Current limiting resistors must be taken into account when designing external analog circuits, as they can slightly change expected results, introducing gain errors, offset, noise, etc.).

**Figure 8:**
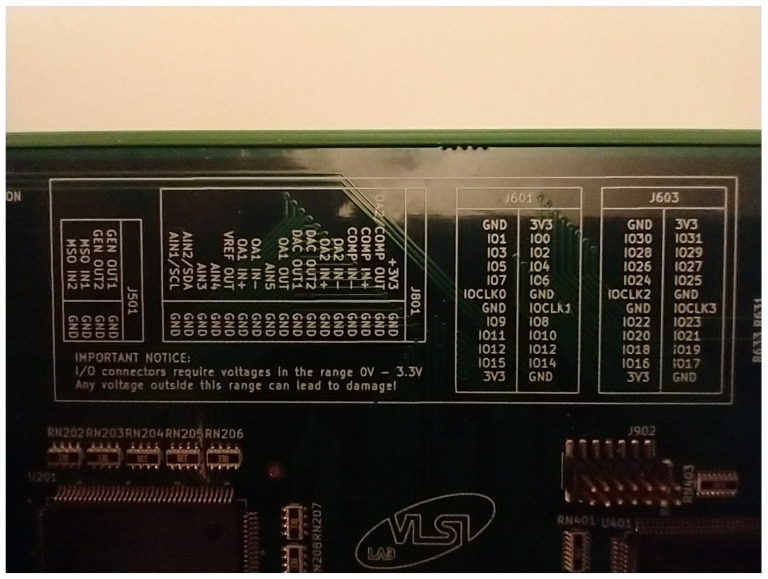
Legend of external I/O connections.

To simplify board usage, given the high number of external signals available on connectors, a legend was inserted into the board silkscreen, showing associations between connector pins and I/O signals. This simple trick should avoid the need to have paper board documentation at hand, and reduces mistakes connecting the board to external circuits. It is also an aid when the student want to use the DSO voltage inputs to debug a circuit, viewing I/O waveforms of the available signals. Figure 8 is a photo of the silkscreen on the real board.

#### 3.5.2. Internal Connections

The following internal configurable connections are present:

- General purpose 32 bit digital I/O bus. It connects external signals to the user FPGA, the user MCU, and the master MCU. The last connection is present only for bits 0–9.
- Double serial channel, from master MCU to user FPGA. Protocols supported are UART, I2C, and CAN.
- QSPI connection, from master MCU to master FPGA.

To avoid device damage due to mistakes in the pin direction configuration, which could cause a short circuit between two outputs, current limiting series resistors are inserted at each FPGA or microcontroller pin.

### 3.6. Power Supply

To minimize costs, it is mandatory to avoid any external power supply. This result can be obtained using USB-C connectors, compatible with USB 3.1 signaling. In fact, this standard allows us to drain up to 3 A @ 5 V from the USB bus used to connect to the host. This means up to 15 W, which is more than sufficient for the board’s needs.

The devices present on the VirtLAB board requires the following power supply rails:

- A 3.3 V @ 2 A: MCU, FPGA I/O, and external connectors;
- A 2.5 V @ 100 mA: FPGA PLL low noise analog supply;
- A 1.2 V @ 2 A: FPGA core.

Starting from the USB 5 V bus voltage, a DC/DC converter built around a Texas Instrument TPS62827 generates the 1.2 V rail for the FPGA core, with a maximum current of 4 A. This rail is particularly sensitive to voltage drops, and the buck converter must be designed to have a very fast transient response. Effective countermeasures are represented by a high switching frequency (2.2 MHz in this case) and by the usage of very-low-ESR multilayer ceramic output capacitors.

An identical circuit, just with different feedback resistor values, generates the 3.3 V power supply voltage with the same maximum current. Even if this power rail would not normally require this high a current level, the 3.3 V is distributed to expansion connectors and can be used to power external circuits. The small money savings of using a different power converter do not compensate for the flexibility gain in having ampere-level currents available for additional circuits.

The 2.5 V low-noise power supply is generated through a low-noise, low-dropout linear regulator (Texas Instruments TPS79325, Dallas, TX, USA), able to source up to 200 mA.

### 3.7. MCU Programming Interface

This part is built up by a commercial module, STLinkV3MODS, produced by STM. It can supply to the board different functions:

- ARM MCU programmer;
- High-speed (15Mb/s) serial interface;
- SPI, I2C, UART, GPIO expansion.

The usage of this kind of module is important for two reasons. First, as an embedded module, without a mechanical box, it is extremely cheap. Second, there is no need to have an external programming tool to allow students to program the user MCU using standard programming tools (STM32CubeIDE), available at no cost.

The main usage of this block is to allow for the loading of user microcontroller firmware developed by the user. Moreover, it allows for user firmware debugging through the gdb tool integrated in STM32CubeIDE.

However, by just adding a simple two-way analog multiplexer between the module and the master or user microcontrollers, it is possible to be able to update the master firmware, too. This capability is very useful, as it allows students to keep even the updated master MCU firmware updated; as an example, when bug fixes are released.

## 4. Conclusions and Future Work

In this work, a custom experimental board has been described that will be used to allow lab access to students of electronics courses in the master degree of Electronics Engineering at Politecnico di Torino. The designed architecture is able to fulfill design requirements, and achieved the following targets:

- Flexibility. Everything is fully programmable, both on the student and on the teacher side. This means that new experiments and new virtual instruments can be freely implemented by just changing the firmware of the MCUs and FPGA configuration.
- Scalability. Devices were chosen to allow for ‘family migration’. As an example, the same footprint can host FPGAs ranging from 6 k to 25 k LEs, and the same applies to MCUs, in which, the same device can be used with different internal memory sizes. This also applies to the HyperRAM, too. This means that a ‘university edition’ of the board, used in campus laboratories, can be built maximizing available hardware resources (and costs), and a ‘student edition’ directly bought by students will be realized with minimum cost hardware.
- Low cost. As the board is specifically designed for teaching, its cost is in the order of USD 70. This is remarkable, as it substitutes an entire set of boards and measurement instruments.

**Figure 9:**
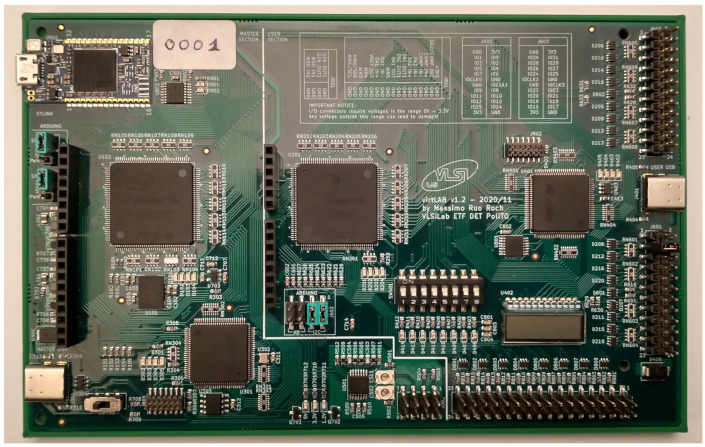
Photograph of the designed board.

The board design is fully completed, two prototypes have been manufactured and are fully working, and 100 boards have been produced, with minor modifications (silkscreen adjustments). Figure 9 is a photo of the final board.

The overall size is 160 × 100 mm (single Eurocard format). Connectors on the right allow us to pick up user area digital signals. The same applies for the big bottom connector, carrying analog signals of the same section. In the upper right corner, the block near the white label is the STLinkV3MODS module.

The board itself was used by students in real courses, starting from November 2021, without any inconvenience. They are:

- Digital integrated systems: Master’s degree in Electronic Engineering, 90 students.
- System level low power techniques for IoT: PhD course in Electrical, Electronics, and Telecommunications engineering, 26 students.

In both courses, VirtLAB boards were given to students, who used them in campus laboratories, in the classroom, and at home. At the end of the semester, boards were returned, and no hardware damage occurred, despite the fact that they were moved around in the backpack.

Unfortunately, there is no possibility of quantitatively comparing students’ feedback about differences between standard laboratories and VirtLAB-based ones. In fact, due to the SARS-CoV2 pandemic, students attending the Digital Integrated Systems master course never participated in real electronics labs in their career (the previous three semesters were held remotely). On the other hand, students of the PhD course expressed positive feedback, and half of them asked to have one VirtLAB board to further implement experiments linked to their research activities.

The flexibility given by the designed architecture allows us to forecast further usages, implementing different virtual instruments, just with firmware and FPGA configuration changes. As stated above, this could be the target of IT courses, too, which were not included while defining system specifications. The same applies for the refinement of the PC software used as the user interface. It will be customized according to course needs. Lastly, at the end of 2019, an IEEE standard was approved, specifically related to virtual laboratories [18], and a possible development is the integration of the developed board to this standard.

## Figures and Tables

**Table 1:** Digital storage oscilloscope input channels.

Input	Bits	Resolution	Gain	Range	Bandwidth
Channel 1	12	0.5 mV	1 V/V	0–2.048 V	1 MHz
Channel 2	12	0.5 mV	1 V/V	0–2.048 V	1 MHz
FPGA 3.3 V current	12	0.022 mA	22 V/A	0–93 mA	150 kHz
FPGA 2.5 V current	12	0.125 mA	4 V/A	0–512 mA	150 kHz
FPGA 1.2 V current	12	0.125 mA	4 V/A	0–512 mA	150 kHz
MCU 3.3 V current	12	0.022 mA	22 V/A	0–93 mA	150 kHz

## Data Availability

Not applicable.
